# Supplementary material for: Evolution uncovers a general tradeoff between recovery after heat shock and growth at elevated temperatures
Source: mBio. 2026 Apr 1;17(5):e03305-25. doi: 10.1128/mbio.03305-25 (PMC13170269; doi:10.1128/mbio.03305-25)
Supplement: Supplemental material — Figures S1 to S9; Tables S1 to S5. [file mbio.03305-25-s0001.pdf]

Supplementary Information

for

Evolution uncovers a general tradeoff between recovery after heat shock and growth at elevated temperatures

Akshat Mall, Katelyn J. Rode, Christopher J. Marx

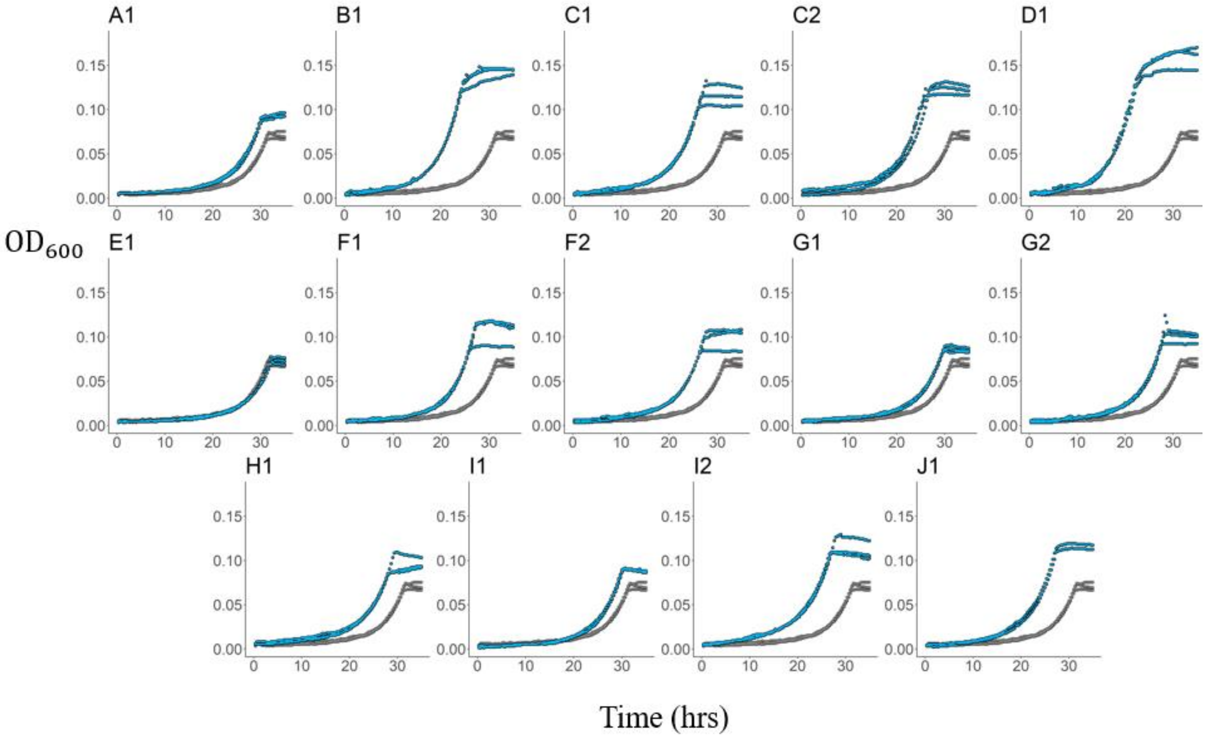

Fig S1. Evolved isolates exhibit improved growth in the evolution regime. Growth curves for all evolved isolates (blue) and ancestor (gray) in the evolution regime of 5 min heat shock at 55 °C followed by recovery on methanol at 30 °C. Three technical replicates shown for each isolate and the ancestor. Note that the difference in final yield is only due to substrate (methanol) evaporation with time.

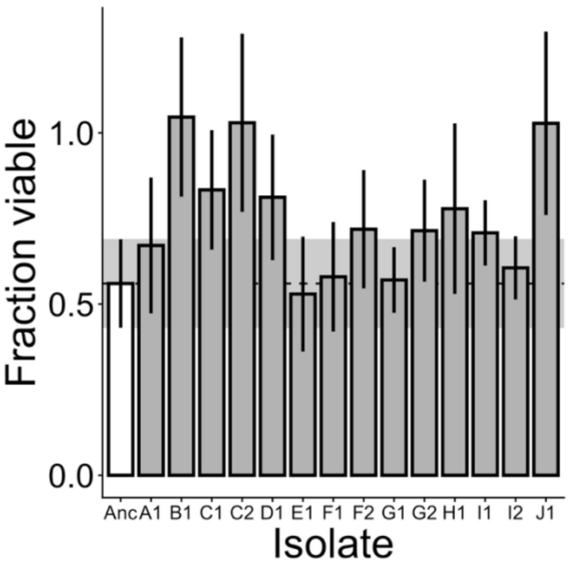

Fig S2. Evolved isolates exhibit improved viability after heat shock. The fraction of viable cells after 5 min heat shock treatment at 55 ˚C was estimated for the ancestor and all evolved isolates. The bars show the mean of 4 replicates, and error bars denote standard deviation. White colored bar represents the ancestor while gray bars represent evolved isolates. Dashed line and shaded region represent mean and mean ± standard deviation for ancestor.

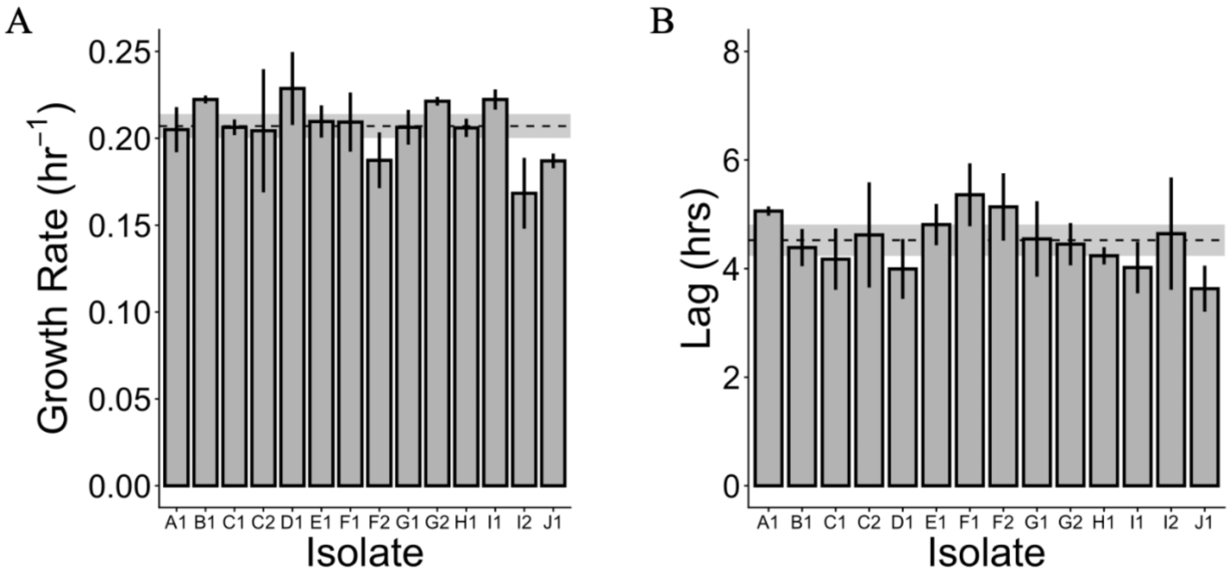

Fig S3. The evolved isolates exhibit no improvement in a normal regime of growth on methanol at 30 ˚C. Specifically, isolates grown on methanol were diluted back into fresh media with methanol at a constant temperature of 30 ˚C, and growth dynamics were quantified. In both panels, bars show the mean of 3 replicates, and error bars denote standard error of mean. Dashed line and shaded region represent mean and mean ± standard error for ancestor. **A)** The change in maximum growth rate is not statistically significant except for isolate I2 (p=0.047) which exhibits a decreased maximum growth rate. **B)** The change in lag time is not statistically significant for any isolate.

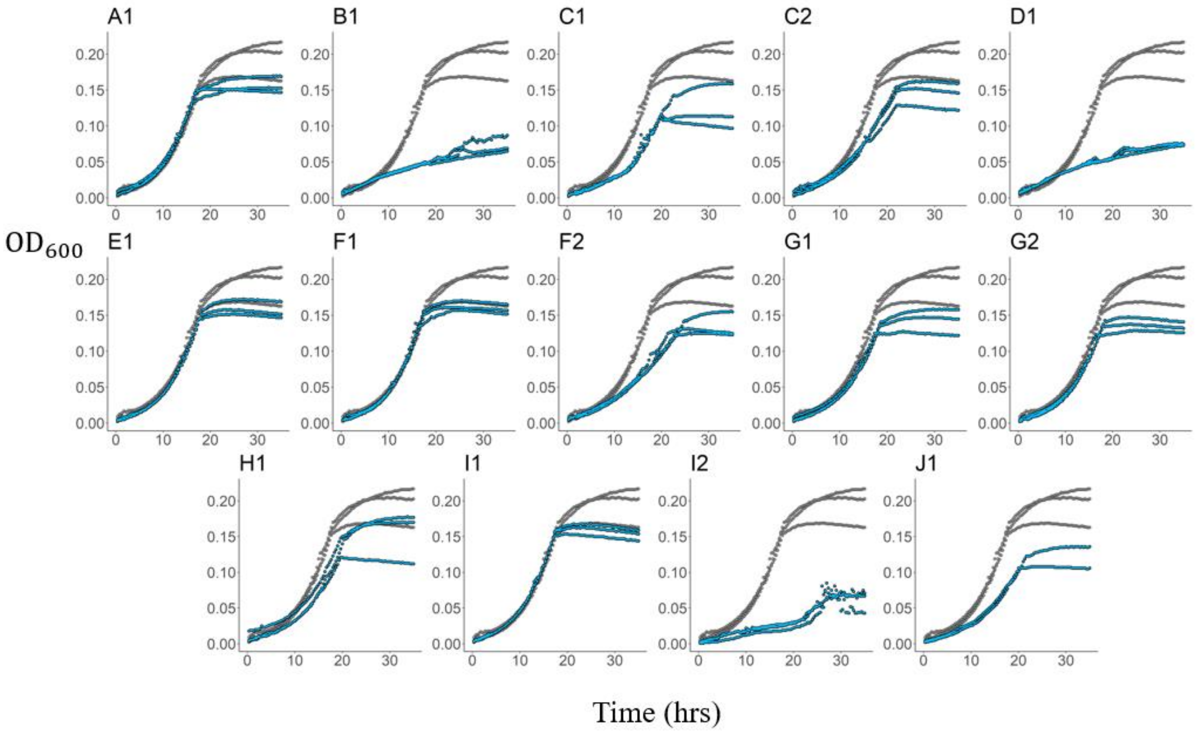

Fig S4. Evolved isolates exhibit growth defects during growth at 35 °C. Growth curves for all evolved isolates (blue) and ancestor (gray) during growth on methanol at 35 °C. Three technical replicates shown for each isolate and the ancestor.

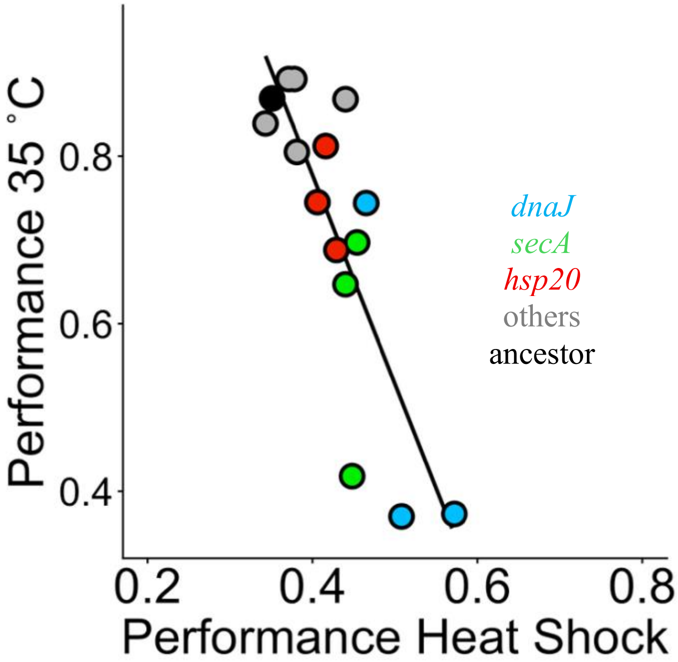

Fig S5. Mutation class explains some of the variance in temperature phenotypes. Performance during growth at 35 ˚C (y-axis) vs performance in a heat shock regime (x-axis) for evolved isolates. Each point represents the mean of three replicates. Points are colored based on the class of mutations in that particular isolate. We focus only on the three most common targets of adaptation – dnaJ (blue), secA (red), hsp20 (green), with the other mutants being gray, and the ancestor in black. All isolates, independent of the class of mutation, exhibit the tradeoff.

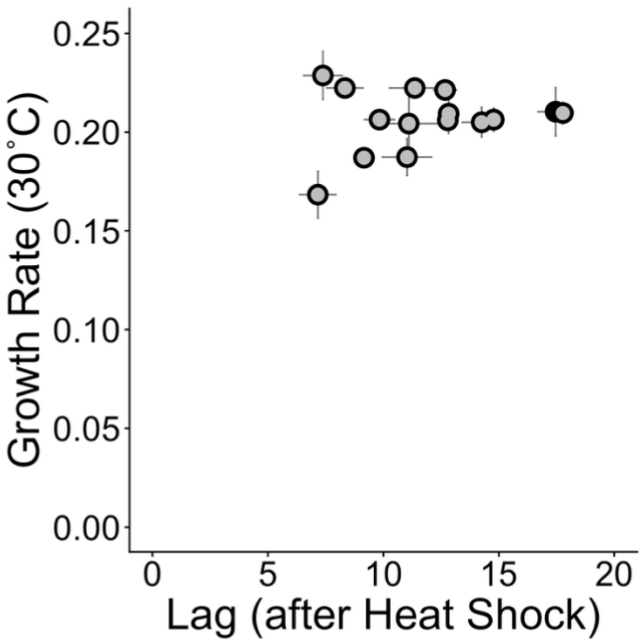

Fig S6. Evolved isolates do not exhibit a tradeoff at the optimal growth temperature of 30˚C. We compared whether improvement in the evolution regime of heat shock was correlated with phenotypic changes during growth in a normal regime of methanol at 30 ˚C. Each point represents the growth rate (hr^{-1}) in a normal regime – methanol at 30 ˚C (y-axis) vs lag time during recovery on methanol at 30 ˚C after heat shock at 55 ˚C for 5 min (x-axis) for an evolved isolate (Table S1). The black shaded point represents the ancestor. Each point represents the mean of three replicates. Error bars represent standard error of mean and are visible when bigger than plotting symbols. All evolved isolates (except isolate E1) exhibit an improvement in lag time after heat shock relative to the ancestor, but they do not show any consistent change in their growth rate at the optimal temperature of 30 ˚C. Only isolate I2 (bottom left point) shows a statistically significant change in growth rate ($p < 0.05$, Student's t-test) and exhibits a reduced growth rate in normal conditions compared to the ancestor. This absence of a tradeoff is in contrast to a strong tradeoff between improvement in a heat shock regime and fitness at a higher temperature of 35 ˚C (Fig 5B in main text).

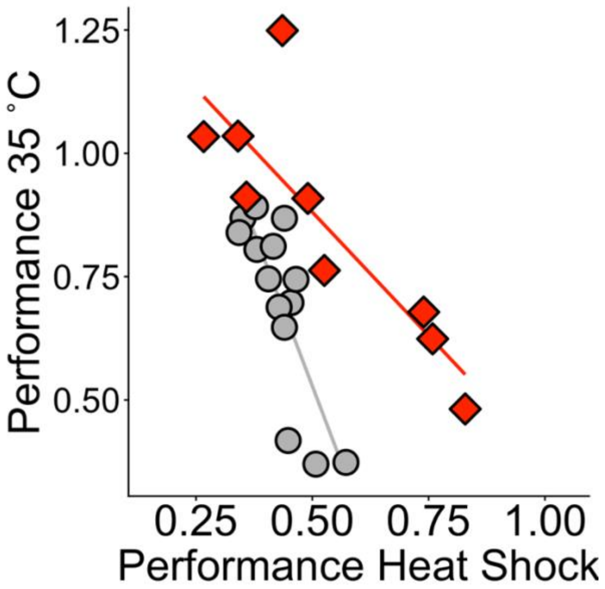

Fig S7. The tradeoff is stronger for the evolved isolates relative to the environmental strains. Each point represents the performance during growth at 35 ˚C (y-axis) vs performance in a heat shock regime (x-axis) for both the evolved isolates (gray) and the environmental strains (red). Lines represent lines of best-fit estimated using linear regression. The strength of the tradeoff as measured by the steepness of the slope of the best-fit lines was significantly greater for the evolved isolates compared to the environmental strains (slopes of -2.5 vs -1.0, $p < 0.01$ using two-way ANCOVA).

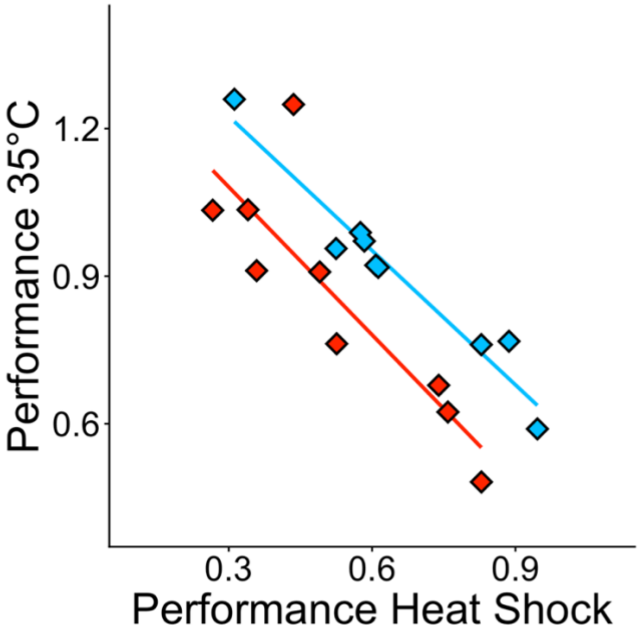

Fig S8. Tradeoff between growth at high temperatures and recovery after heat shock for environmental isolates is independent of carbon source. Each point represents the performance at an elevated temperature of 35 ˚C (y-axis) vs the performance during recovery at 30 ˚C after heat shock at 55 ˚C for 5 min (x-axis). We define performance as the time to reach a threshold OD in each regime relative to the time taken at an optimal temperature of 30 ˚C (Please see Fig 5A for more details on the metric). Points colored red denote results with methanol as the carbon source (same as Fig 5C). Points colored blue denote results with succinate as the carbon source. Each point represents the mean of 4 replicates. Lines represent the line of best fit for each substrate.

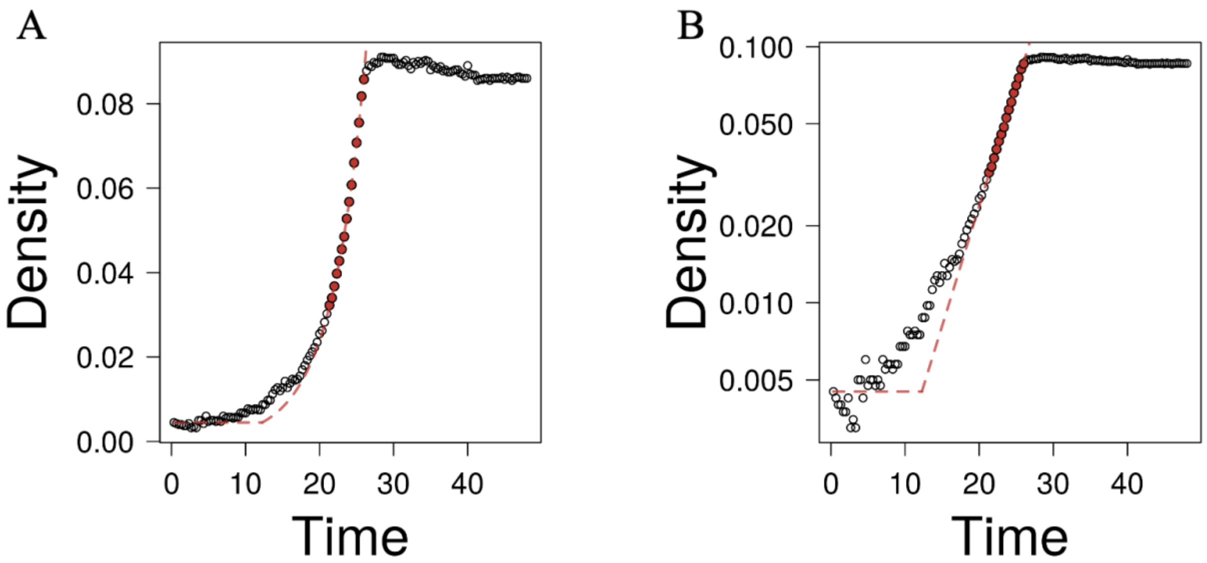

Fig S9. Growth rates and lag times were quantified by fitting a line to log-transformed Optical Density vs time data. Example plots of analysis shown here for one replicate of isolate F1 during recovery after heat shock. For illustration purposes, we show both **A)** regular scale, and **B)** OD axis being log-transformed. The points shaded red represent the points used to estimate maximum growth rate, and the red dashed line indicates the line of best fit for the red points, whose slope represents the maximum growth rate. The point on the time axis where this line intersects with a horizontal line from the starting OD represents the lag time. In this example, our estimated lag time = 12.26 h, and maximum growth rate = 0.216 h^{-1}.

Isolate	Annotation	Gene	Mutation	Change	Location
A1	×	×	×	×	×
B1	preprotein translocase, SecA subunit	Mext_1478	G → A	G144D (GGC→GAC)	1655946
C1*	short-chain dehydrogenase/ reductase SDR	Mext_1019	A → C	T220P (ACC→CCC)	1105714
	chaperone protein DnaJ	Mext_2961	T → G	Y129D (TAC→GAC)	3309193
	diguanylate cyclase	Mext_3867	A → T	M589K (ATG→AAG)	4294005
C2	preprotein translocase, SecA subunit	Mext_1478	Δ1 bp	coding (2828/2913 nt)	1658343
D1	preprotein translocase, SecA subunit	Mext_1478	G → T	D61Y (GAC→TAC)	1655696
E1	N/A	Mext_3633/ Mext_3634	A → G	intergenic	4011581
F1	flagellar FlbT family protein	Mext_1248	Δ1 bp	coding (345/417 nt)	1384271
F2*	AFG1–family ATPase	Mext_0934	T → C	V239A (GTC→GCC)	1011093
	heat shock protein Hsp20	Mext_4556	A → G	F16L (TTC→CTC)	5086575
G1	3',5'-cyclic-nucleotide phosphodiesterase	Mext_1789	Δ171 bp	coding (86-256/279 nt)	2008936
G2	N/A (7 bp upstream of heat shock protein Hsp20)	Mext_4262/ Mext_4263	T → A	intergenic	4744167
H1	heat shock protein Hsp20	Mext_1620	G → T	K141N (AAG→AAT)	1807080
i1	SpoVR family protein	Mext_2061	G → C	G94A (GGC→GCC)	2296689
i2	Chaperone protein DnaJ	Mext_2961	Δ6 bp	coding (586-591/1158 nt)	3309394
J1*	N/A (32 bp upstream of luciferase family protein)	Mext_1738/ Mext_1739	$(CTCG)_{6\rightarrow7}$	intergenic	1961229
	chaperone protein DnaJ	Mext_2961	G → A	G307D (GGT→GAT)	3309728

Table S1. Mutations identified in evolved isolates. Isolate name denotes replicate information (A-J), and the number (1 or 2) designated to the isolate picked for that replicate. E.g. – C1 and C2 denote different individuals from the same replicate in the evolution experiment. Isolates with multiple mutations denoted with asterisks.

**Strain**	**Growth rate (hr^{-1})**	***p*-value (growth rate)**	**Lag (hrs)**	***p*-value (lag)**
Ancestor	0.207±0.012	NA	17.7±1.4	NA
A1	0.188±0.007	0.09	14.6±1.1	0.041*
B1	0.206±0.003	0.83	8.4±1.2	0.001*
C1	0.191±0.003	0.14	9.5±1.1	0.002*
C2	0.202±0.008	0.56	11.4±2.4	0.027*
D1	0.236±0.017	0.08	7.8±1.3	0.001*
E1	0.199±0.024	0.61	18.1±0.7	0.699
F1	0.220±0.007	0.20	12.0±2.0	0.021*
F2	0.194±0.006	0.19	11.1±1.8	0.008*
G1	0.188±0.005	0.09	14.8±0.3	0.065
G2	0.210±0.022	0.88	13.0±1.1	0.011*
H1	0.179±0.002	0.06	13.0±0.4	0.021*
I1	0.203±0.007	0.64	11.4±1.9	0.011*
I2	0.168±0.003	0.02*	7.4±1.4	0.001*
J1	0.183±0.006	0.06	9.2±0.2	0.007*

Table S2. Growth rate and lag comparisons between ancestor and heat shock evolved isolates after heat shock treatment. Growth rate and lag values represent the mean of 3 replicates ± standard error of mean. *p*-values are based on a comparison of the evolved isolates and the ancestor using the Student's t-test. The statistically significant points ($p < 0.05$) are marked with an asterisk (*).

Isolate	Mutation	Performance Heat Shock	Performance 35°C
A1	×	0.379±0	0.892± 0.024
B1	preprotein translocase, SecA subunit	0.508±0	0.370±0.083
C1*	short-chain dehydrogenase/ reductase SDR	0.454±0.036	0.697± 0.01
	chaperone protein DnaJ		
	diguanylate cyclase		
C2	preprotein translocase, SecA subunit	0.465±0.011	0.744±0.01
D1	preprotein translocase, SecA subunit	0.572±0.058	0.373±0.026
E1	N/A	0.343±0.041	0.839±0.025
F1	flagellar FlbT family protein	0.44±0	0.868±0
F2*	AFG1–family ATPase	0.429±0	0.688±0.024
	heat shock protein Hsp20		
G1	3',5'-cyclic-nucleotide phosphodiesterase	0.381±0.025	0.805±0.02
G2	N/A (7 bp upstream of heat shock protein Hsp20)	0.416±0.03	0.812±0.012
H1	heat shock protein Hsp20	0.406±0.029	0.745±0.036
i1	SpoVR family protein	0.378±0.025	0.892±0
i2	Chaperone protein DnaJ	0.448±0.035	0.418±0.007
J1*	N/A (32 bp upstream of luciferase family protein)	0.440±0.083	0.647±0.018
	chaperone protein DnaJ		
Anc	×	0.351±0	0.869±0.023

Table S3. Performance of evolved isolates and ancestor in a heat shock regime and during growth at 35 °C. The tradeoff between recovery after heat shock and growth at 35 °C is not limited to mutations in a specific locus but is a general phenomenon. Values represent mean performance of 4 replicates ± standard deviation.

Background Strain	Barcoded Strain	Environmental source
M. extorquens PA1[1] (CM2730)[2]	CM5201	*Arabidopsis thaliana*
M. nodulans ORS 2060[3]	CM5156	Root nodules of *Crotalaria*
M. extorquens AM1[4] (CM2720)[2]	CM5162	Airborne contaminant
M. extorquens CM4[5]	CM5151	Soil at a petrochemical factory
SLI_158*	CM5140	Soybean leaf
SLI_210*	CM5141	Soybean leaf
TLI_801*	CM5149	Tomato leaf
TLI_802*	CM5150	Tomato leaf
CM6257 [#]	n/a	Western Red Cedar leaf

Table S4. List of strains used to study the universality of the tradeoff (Fig 5C in main text). All strains except CM6257 have a neutral DNA barcode inserted into the chromosome, along with a Kanamycin resistance cassette (Alexander B. Alleman, Monica J. Pedroni, Galen Beery, CJM, unpublished).

1. Knief, C., Frances, L. & Vorholt, J. A. Competitiveness of Diverse *Methylobacterium* Strains in the Phyllosphere of *Arabidopsis thaliana* and Identification of Representative Models, Including *M. extorquens* PA1. *Microb Ecol* **60**, 440–452 (2010).
2. Delaney, N. F. *et al.* Development of an Optimized Medium, Strain and High-Throughput Culturing Methods for *Methylobacterium extorquens*. *PLOS ONE* **8**, e62957 (2013).
3. Jourand, P. *et al. Methylobacterium nodulans* sp. nov., for a group of aerobic, facultatively methylotrophic, legume root-nodule-forming and nitrogen-fixing bacteria. *International Journal of Systematic and Evolutionary Microbiology* **54**, 2269–2273 (2004).
4. Peel, D. & Quayle, J. Microbial growth on C1 compounds. 1. Isolation and characterization of Pseudomonas AM 1. *Biochemical Journal* **81**, 465–469 (1961).
5. McDonald, I. R., Doronina, N. V., Trotsenko, Y. A., McAnulla, C. & Murrell, J. C. *Hyphomicrobium chloromethanicum* sp. nov. and *Methylobacterium chloromethanicum* sp. nov., chloromethane-utilizing bacteria isolated from a polluted environment. *International Journal of Systematic and Evolutionary Microbiology* **51**, 119–122 (2001).

* Collen Friel and N. Cecilia Martinez-Gomez, unpublished

[#] Noah M. Arts, Alexander B. Alleman, and CJM, unpublished.

Chromosome position	Mutation	Annotation	Gene
1,099,002	$(C)_{5\rightarrow4}$	Intergenic (+63 / +28)	Mext_1011 / Mext_1012
1,289,299	Δ1 bp	Intergenic (-281 / -257)	Mext_1169 / Mext_1170
1,520,170	Δ7108 bp	cellulose biosynthesis deletion	Mext_1367 – [Mext_1370]
1,527,312	C → T	D239D (GAC → GAT)	Mext_1370
1,690,785	Δ1 bp	Intergenic (-1367 / +961)	Mext_1510 / Mext_1512
3,838,949	Δ1 bp	Intergenic (-15 / -1373)	Mext_3458 / Mext_3460

Table S5. Mutations in WT ancestor (lab strain CM2730) compared to NCBI reference genome of *M. extorquens* PA1.
